# A Chinese girl of Blau syndrome with renal arteritis and a literature review

**DOI:** 10.1186/s12969-023-00804-z

**Published:** 2023-03-13

**Authors:** Qiaoqian Zeng, Haimei Liu, Guomin Li, Yifan Li, Wanzhen Guan, Tao Zhang, Yinv Gong, Xiaomei Zhang, Qianying Lv, Bingbing Wu, Hong Xu, Li Sun

**Affiliations:** 1 Department of Rheumatology, Children’s Hospital of Fudan University, Shanghai, China; 2 Medical Transformation Centre, Children’s Hospital of Fudan University, Shanghai, China; 3 Department of Rheumatology, National Children’s Medical Center, Children’s Hospital of Fudan University, 399 Wan Yuan Road, Shanghai, 201102 People’s Republic of China

**Keywords:** Blau syndrome, Vasculitis, Renal arteritis, Medium-and large-vessel arteritis

## Abstract

**Background:**

Blau syndrome is a rare autoinflammatory disease caused by autosomal dominant mutations in the CARD15/NOD2 gene. Vascular involvement is a rare phenotype in Blau syndrome patients. In this study, we aimed to describe a 20-year- old Chinese girl with Blau syndrome complicated by renal arteritis. In addition, we summarized a literature review of published cases of vascular involvement in patients with Blau syndrome.

**Case presentation:**

We describe a 20-year-old girl who was initially misdiagnosed with juvenile idiopathic arthritis (JIA) almost 15 years prior. In October 2019, she developed renal arteritis at the age of 17 years and was eventually diagnosed with Blau syndrome. A de-novo M513T mutation was found in her gene testing. A review of the literature on patients with Blau syndrome and vasculitis showed that a total of 18 cases were reported in the past 40 years. The vast majority of them were predominantly involved medium and large vessel arteritis. Of the 18 patients included in our literature review, 14 patients had aorto-arteritis, and 4 of them had renal artery involvement. Two patients presented with renal artery stenosis, 1with a sinus of Valsalva aneurysm, and 1 with retinal vasculitis.

**Conclusion:**

A detailed medical history inquiry and a careful physical examination are helpful for the early identification of Blau syndrome, especially for infant onset refractory JIA. Medium-and large-vessel arteritis is a rare clinical manifestation in Blau syndrome patients. Careful examination of the peripheral pulse and measurement of blood pressure at every regular visit may be helpful in the early identification of Blau syndrome-arteritis. Early diagnosis and appropriate treatment may prevent or delay the occurrence of severe symptoms in patients to improve the patient’s quality of life.

**Supplementary Information:**

The online version contains supplementary material available at 10.1186/s12969-023-00804-z.

## Introduction

Blau syndrome (BS), first described by Edward Blau in 1985, is a rare autosomal dominant, granulomatous, autoinflammatory disease caused by mutations in the NOD2/CARD15 gene [1, 2]. The disorder is characterized by early onset and the triad of noncaseating granulomatous uveitis, arthritis, and skin rashes. Skin manifestations are usually the earliest symptoms to appear in children younger than 5 years of age. The most common skin lesions are scaly erythematous plaques and multiple subcutaneous nodules without obvious subjective symptoms [3]. Histology of the lesions shows noncaseating granulomas with multinucleated giant cells [4]. Joint manifestations often appear in the first 10 years of life [5]. In the absence of uveitis or skin findings, the initial manifestation of arthritis is often mistaken for juvenile idiopathic arthritis (JIA). Joint involvement usually presents as symmetrical polyarthritis, which may lead to deformity of joints. Ocular lesions also require the closest attention and can affect the quality of life. Most commonly, patients develop recurrent, granulomatous uveitis in both eyes, including anterior uveitis and panuveitis. In addition to the typical clinical manifestations, other organs can be affected including medium and large vessel vasculitides.

Here, we described a girl with symptomatic renal arteritis against the background of Blau syndrome with an M513T de-novo variant in NOD2. Additionally, we also reviewed the clinical manifestations of the cases of Blau vasculitis published before June 2022.

## Case description

A girl, now 20 years old, presented to our hospital for the first time at the age of 7 years and 8 months, with multiple-joint swelling and limited motion for more than 5 years and recurrent fever for 18 months. Her long-term history of arthritis could be traced back to when she started to suffer from symmetrical polyarthritis at the age of 2 years, and her arthritis was in both knees, both wrists, the metacarpophalangeal joints, the proximal and distal phalangeal joints. She presented to the pediatric doctors in the local hospital by her parents and was diagnosed with juvenile idiopathic arthritis (JIA). The doctor treated her mostly with nonsteroidal anti-inflammatory drugs (NSAIDs) and methotrexate (MTX). During the following 5 years, her symptoms were not well controlled. At the age of 6 years, she had repeated and unexplained fevers. Anti-infection and symptomatic treatment were given but were ineffective. She was taken to her local pediatrician many times and received steroid treatment for several months. When she was 7 years of age, she complained of attacks of redness in both eyes but denied any history of vision loss. She was diagnosed with anterior uveitis of both eyes and her ocular symptoms disappeared after receiving ophthalmic treatment (tobramycin dexamethasone eye drops). Regarding her complete past medical history, in addition to the above manifestations, she had a squamous rash once at 10 months of age, which was ignored by her parents at that time.

**Fig. 1:**
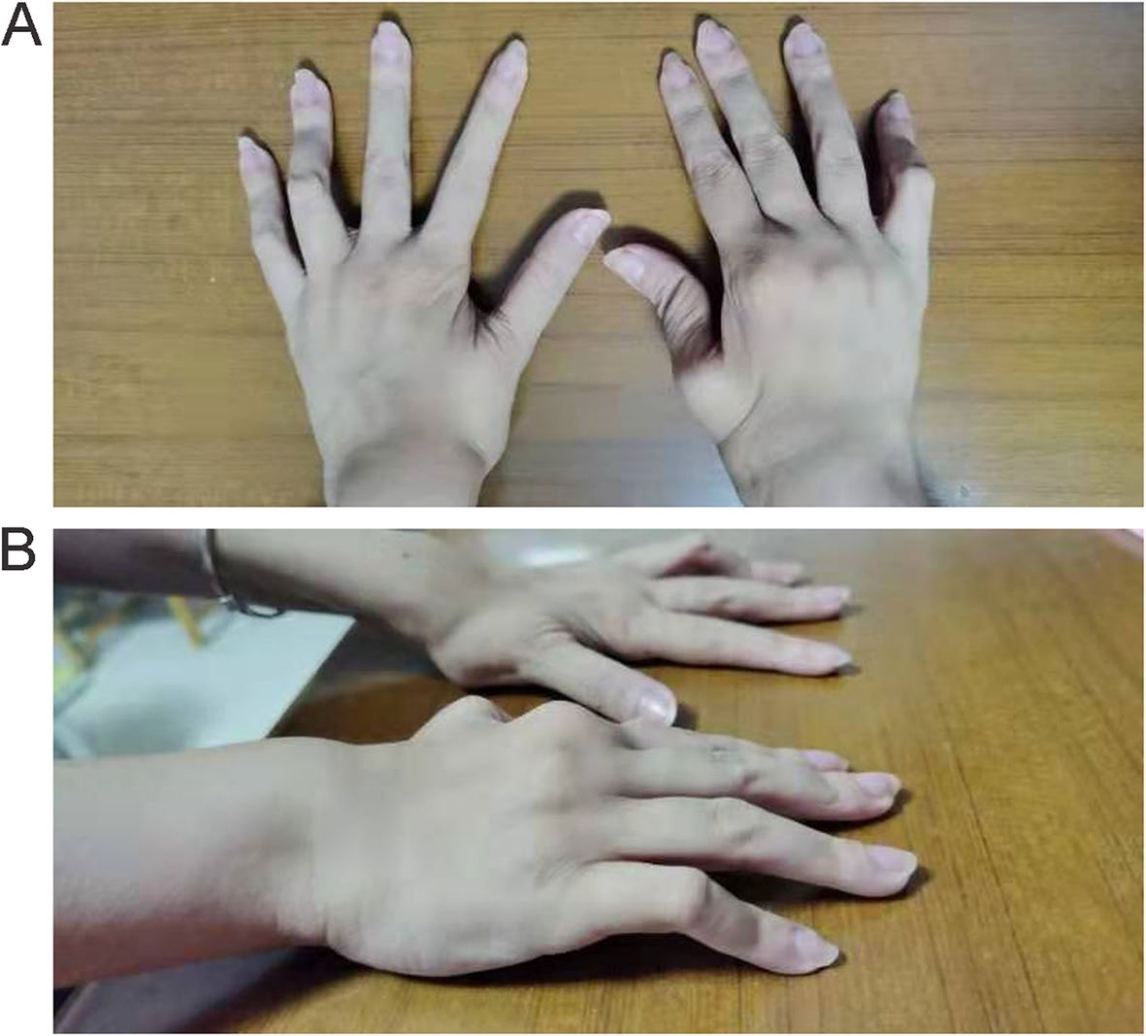
**A**, **B** Clinical manifestations of the patient. Swan neck deformity of all fingers

In August 2009, when she was 8 years and 9 months old, the girl was referred to our hospital for the first time regarding recurrent arthritis in the absence of ocular complaints, rash and other symptoms. There was no other contributory past medical or history or family history. Physical examination revealed swan neck deformities of all fingers (Fig. 1A, B**)**, limited joint mobility, swelling of the wrists, elbows, and knees, and a positive right 4-word test. No other remarkable findings were observed on physical examination. Blood tests showed a significant increase in the C-reactive protein (CRP) level and erythrocyte sedimentation rate (ESR). Her rheumatoid factor and human leukocyte antigen B27 (HLA-B27) were negative. No positive findings were found on her ophthalmic examination. MRI and other imaging examinations supported the manifestations of arthritis. With a diagnosis of JIA (polyarticular, RF negative), treatment was initiated and consisted of Etanercept, recombinant human tumor necrosis factor-Fc (rhTNFR:Fc), 0.8 mg/kg per week combined with MTX and NSAIDs. She showed improvement in her joint swelling and fever, but these symptoms still had recurred. In the following 10 years, the patient was followed up irregularly, and she had stopped etanercept on her own. Between 2015 and 2019, she was intermittently treated with prednisolone at doses ranging from 2.5 mg to 10 mg per day. Regrettably, the girl had vision loss in both eyes in 2018. An ocular examination showed obsolete iridocyclitis in both eyes and left eye cataracts. She underwent left eye cataract surgery in May 2018 in the Ophthalmology & Otorhinolaryngology Hospital, and her visual acuity recovered after the operation. In October 2019, she developed unexplained hypertension, which was found on a routine physical examination at school. She was referred to the local hospital and given nifedipine, but her blood pressure was not controlled.

**Fig. 2:**
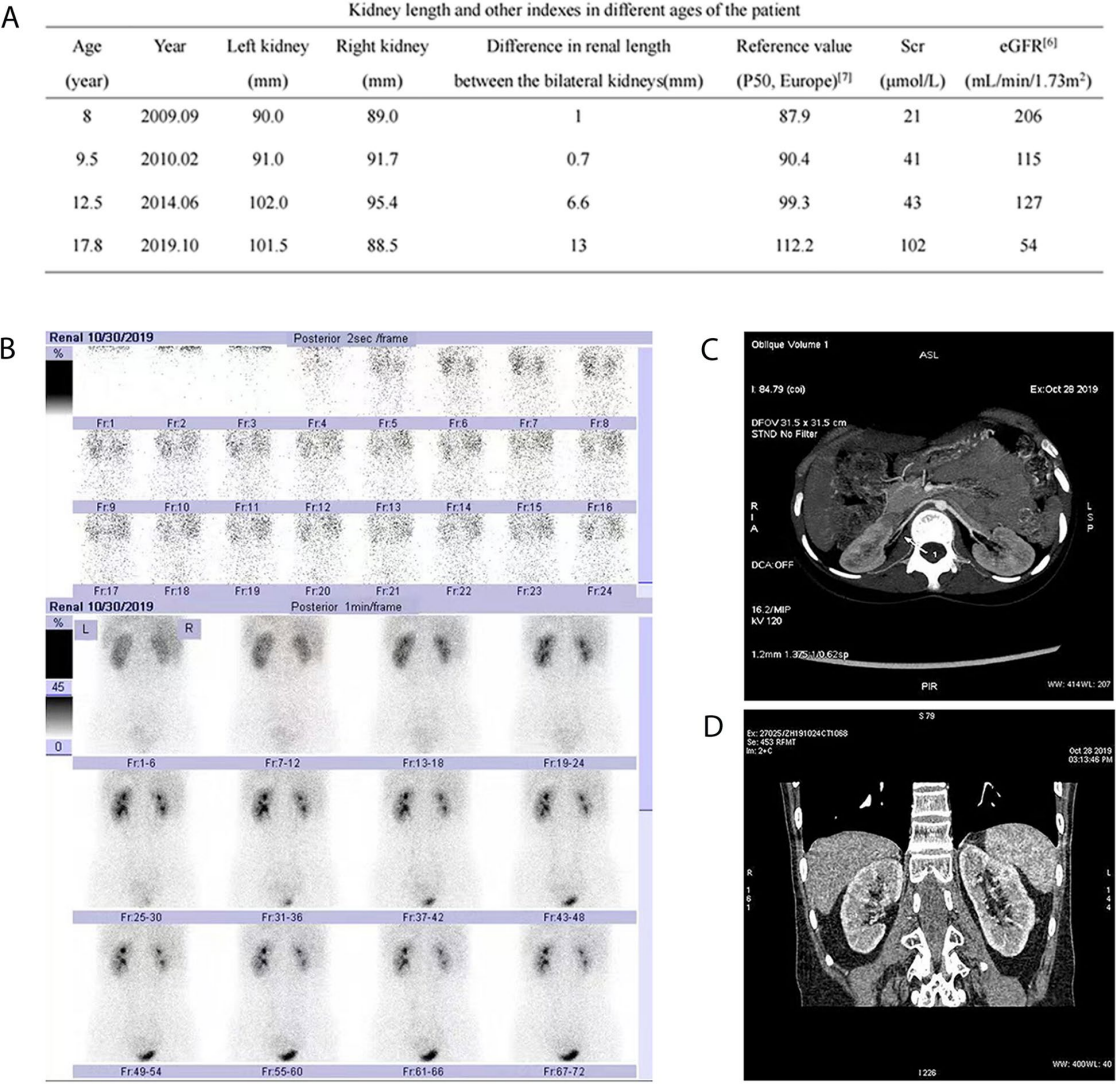
**A** Kidney length and other indexes in different ages of the patient. **B** 99mTc-DTPA (October 30, 2019) renography demonstrated that the right kidney was smaller than the left kidney with poor renal perfusion and impaired renal function (right kidney 41.4%, the left kidney 58.6%). The GFR [ml·min-1·(1.73 m2)-1] of the kidneys was 74.4 (right kidney 30.8, left kidney 43.6). **C**. Computed tomography angiography (CTA) (October 28, 2019) of the renal artery illustrated that the branches of the renal artery in the right hilum had mild stenosis. **D** CTA showed that the right kidney was smaller than the left kidney

On October 24, 2019, the girl was transferred to our hospital. Physical examination revealed that her blood pressure (BP) was 140/70 mmHg in the left upper limb, 139/79 mmHg in the right upper limb, 167/76 mmHg in the left lower limb, and 165/67 mmHg in the right lower limb. Before this, her blood pressure was increased once in a visit in 2017 in the only few outpatient visits, and was normal at other times. Moreover, she had painless fixed flexion deformities of multiple joints on physical examination. Additionally, ophthalmologic checks showed bilateral band-shaped degeneration of the cornea, with a corrected visual acuity of 0.4 in the right eye (OD) and 0.3 in the left eye (OS). She was diagnosed with bilateral panuveitis by an ophthalmologist. Various laboratory tests were ordered, and the results were as follows: her 24-h urinary protein excretion was 0.19 g and her serum creatinine was 102 μmol/L. We collected data on the serum creatinine (Scr) and eGFR [6] of the patient during the past 10 years (Fig. 2A). According to the results, her Scr began to increase (ranging from 75 to 92 μmol/L) in approximately 2017 and finally increased to 102 μmol/L in October 2019. Her renin–angiotensin–aldosterone, CRP, ESR and thyroid hormone were within the normal ranges. Her autoantibodies were negative. Ambulatory blood pressure monitoring (ABPM) showed that the 24-h average BP was 143/83 mmHg (> 130/80 mmHg), the daytime average BP was 144/86 mmHg (> 135/85 mmHg) the and nighttime mean BP was 140/76 mmHg (> 120/70 mmHg).

B ultrasound demonstrated that the right kidney (8.85*3*3.42 cm) was smaller than the left kidney (10.2*3.86*4.05 cm). The difference in length between the two kidneys was 1.35 cm. We searched and summarized all the renal B ultrasound data of the patient in the past 10 years compared with kidney length normative values in children [7] (Fig. 2A). The sizes of the kidneys did not increase with age, the right kidney gradually decreased in size, and the size difference of both kidneys gradually increased. 99mTc-DTPA renography demonstrated that the right kidney was smaller than the left kidney with poor renal perfusion and impaired renal function (Fig. 2B). The GFR [ml·min-1·(1.73 m2)-1] of the kidneys was 74.4 (right kidney 30.8, left kidney 43.6). A significant difference was observed in the two splits of renal function: the renal function was 41.4% in the right kidney and was 58.6% in the left kidney. Computed tomography angiography (CTA) of the renal artery illustrated that the branches of the renal artery in the right hilum had mild stenosis (Fig. 2C), and the right kidney was smaller than the left kidney (Fig. 2D). No significant abnormalities were observed in the thoracic aorta, abdominal aorta, or cerebrovascular system. No remarkable findings were revealed on echocardiography and during an electrocardiogram. To further clarify the situation of the patients’s renal vessels, we also suggested performing digital angiography. However, her parents refused this invasive examination. The patient was diagnosed with right renal vasculitis through CTA of the renal artery (mild stenosis in the branch of the renal artery of the right renal hilus) and 99mTc-DTPA renography (difference in bilateral renal function and GFR) based on her two unequally sized kidneys (the gradually shrinking right kidney and the normal left kidney).

**Fig. 3:**
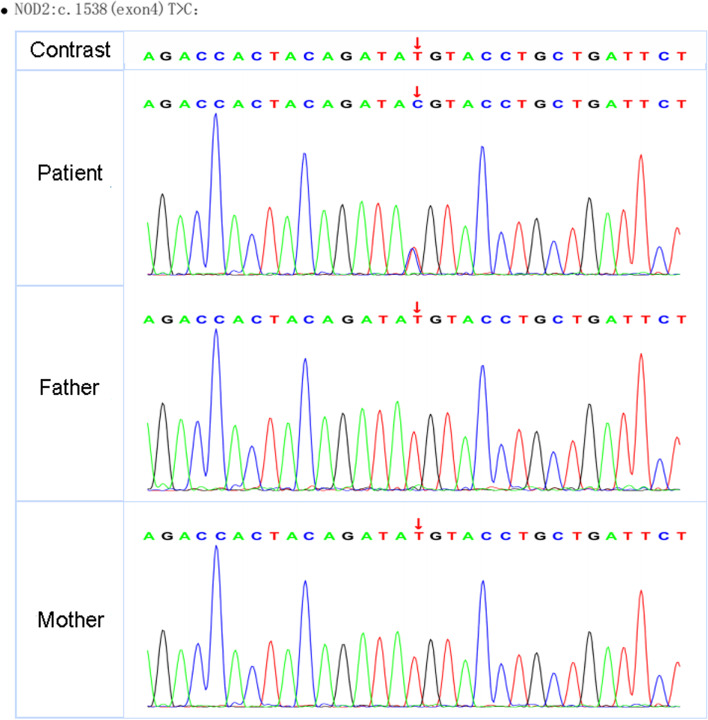
Gene sequencing of the NOD2 gene. The patient had a de-novo heterozygous mutation in the NOD2 gene (c.1538 T > C, *p*.M513T). The gene analysis of her parents was normal

In view of the clinical manifestations mentioned above, including recurrent polyarthritis, fever, bilateral panuveitis and kidney involvement in medium-vessel vasculitis, it was obvious that this girl might in fact not have JIA. The diagnosis was likely an autoinflammatory disease. Thus, we performed whole genome sequencing (WGS) for the patient and her family. The results revealed that she had a de-novo heterozygous mutation in the NOD2 gene (c.1538 T > C, p.M513T) (Fig. 3), with her parents presenting with the wild type. The M513T (1538 T/C) mutation was a heterozygous mutation and has been reported previously. According to the presentation and genetic test results, she was finally diagnosed with Blau syndrome and renovascular hypertension.

Considering her ocular lesions and unsatisfactory therapeutic effect, we suggested treating her with an anti-TNF-α monoclonal antibody (adalimumab) based on the previous studies [3]. However, the patient refused the treatment because of financial reasons. Finally, she temporarily accepted a low dose of oral prednisolone, MTX, metoprolol and plendil. Her BP was gradually decreased and was controlled at approximately 130/80 mmHg. The OD and OS corrected vision were gradually restored to 0.6 after approximately 3 months. Her serum creatinine level slightly decreased and remained between 80–90 μmol/L after nearly 3 months of follow-up. In February 2020, she was referred to an adult hospital after reaching the age of 18. In the latest telephone consultation with the patient in December 2022, she stated that she was receiving prednisolone (15 mg/day) and tofacitinib (5 mg bid), and MTX had been discontinued because of abnormal transaminase. There were no new symptoms in the joints or eyes. There were also no rashes. Hematology examinations indicated that the patient’s ESR (25 mm/h) and serum creatinine (138 μmol/L) had increased. Her blood pressure was maintained at approximately 140/90 mmHg without any antihypertensive therapy.

## Literature review

A literature search for children with Blau syndrome and vasculitis was carried out using the PubMed database until June 2022. The search was performed using the following keywords or MeSH terms: “Blau syndrome", "Early-onset sarcoidosis (EOS)”, "vascular involvement", "vasculitis”, "arteritis”, and "vessel". There were few epidemiological data, and the literature was nearly limited to some case reports. Finally, we identified 12 articles describing 20 patients with Blau syndrome and vasculitis in total during the past 40 years. Two articles were excluded because the patients who were mentioned overlapped. Therefore, 10 articles including 18 patients were included and analyzed in our study. As shown in Table 1 [8, 9, 10, 11, 12, 13, 14, 15, 16, 17], 18 patients were reported from 1982 to 2017 and were among 5 different countries. According to these studies, most of the patients had the typical triad of Blau syndrome consisting of arthritis, rash, and uveitis. Arthritis was the most frequent clinical feature, with a frequency of 100%. Due to inadequate information, the incidence of uveitis and rash in these patients was not determined. Fourteen patients had aorto-arteritis, and four of them also had renal artery involvement. Of the remaining four patients, two had renal arteritis, one had a sinus of Valsalva aneurysm [13], and one had retinal vasculitis [16]. Among the reports containing complete gene testing information of the patients, we found three various mutations, R334W, G464W, and D382E, in the NOD2 gene. In our center, the patient in the case report we described above had an M513T mutation.

**Table 1:** Review of published cases with pediatric Blau syndrome and vasculitis

Year,author	Country	No of cases /gender	Blau manifestations			Vascular Phenotype	Substitution
			R	A	U		
1982,Rotenstein et al. [8]	America	2/F	^a^	^a^	^a^	Splenic arteritis, Renal arteritis, Iliac arteritis/Aortitis	NA
1986,Gross et al. [9]	Canada	1/F	^a^	^a^	^a^	Right renal artery stenosis, Right superior mesenteric artery stenosis	NA
1990, CD rose et al. [10]	America	1/NA	^a^	^a^	^a^	Takayasu arteritis	NA
1996, Gedalia et al. [11]	America	1/M			^a^	abdominal aortic aneurysm	NA
2002,Wang et al. [12]	America	1/M		^a^	^a^	large-vessel	R334W
2010,Mourad et al. [13]	England	1/F	NA	NA	NA	Sinus of valsalva aneurysm	NA
2012,Khubchandani et al. [14]	America	1/F		^a^		Takayasu arteritis ( stenosis of thoracic aorta,left common carotid artery, left renal artery)	G464W
2013,Yuzaburo Inoue et al. [15]	Japan	1/M	^a^	^a^	^a^	Takayasu arteritis (stenosis of the left middle cerebral artery, aortitis,bilateral renal artery stenosis)	D382E
2013,Sejal R Amin et al. [16]	America	1/M				Retinal Vasculitis	NA
2017,Li et al. [17]	China	8/NA	NA	^a^	NA	Aorto-arteritis	NA
2022,Our medical center	China	1/F	^a^	^a^	^a^	Right renal artery stenosis	M513T

*F* Female, *M* Male, *R* Rash, *A* Arthritis, *U* Uveitis, *NA* Not available

^a^means that the patient had this clinical manifestation

## Discussion

Blau syndrome and early onset sarcoidosis (EOS) are the familial and sporadic forms, respectively, of the same disease. Now, they are uniformly called Blau syndrome [18]. The incidence of the disease is unknown due to the lack of related research. To our knowledge, there have been at least more than 200 persons with Blau syndrome worldwide to date [17].

It is an autosomal dominant inherited disease resulting from mutations in the NOD2/CARD15 gene. The CARD15/NOD2 gene is located in chromosome region 16q12.1–13 [19]. This gene encodes nucleotide oligomerization domain 2 (NOD2), which is a multidomain protein of 1040 amino acids. NOD2, a member of the nucleotide oligomerization domain (Nod)-like receptor (NLR) family, functions as an intracellular pattern recognition receptor (PRR) for muramyl dipeptide (MDP), which stems from peptidoglycan (PGN) of both gram-negative and gram-positive bacteria [20]. The NOD2 protein is composed of three domains: caspase activation and recruitment domains (CARDs), nucleotide-binding oligomerization domain (NOD) (also called NACHT), and leucine-rich repeat (LRR) [21]. NOD2 plays an important role in regulating the NF-kB and MAPK signaling pathways to regulate the release of downstream inflammatory factors [22]. Mutations in the NOD2 gene are closely related to two distinct granulomatous diseases: Crohn's disease (CD) and Blau syndrome. Between the two different diseases, CD-related variants are loss-of-function mutations and mainly exist in LRR, while Blau syndrome-related mutations are gain of function mutations and occur in the NACHT domain [23]. NOD2 is mainly expressed in the cytoplasm of monocytes [24], dendritic cells [25], and Paneth cells [26], which are the main components of noncaseating granulomas. However, the pathogenesis is still not clearand requires further exploration and research.

Blau syndrome is characterized by the triad of granulomatous arthritis, uveitis, and skin rashes [12]. In pediatric populations, the clinical manifestations of the triad mostly appear progressively over time, even at intervals of several years [27]. Among these symptoms, skin involvement was the first clinical symptom of the classical Blau syndrome triad and occurred at a median age of 2.25 years in the patients described in the literature [28, 29, 30]. Skin lesions often lack typical clinical characteristics and can manifest as papules, nodules, ichthyosiform lesions, or other various skin lesions [30]. Sometimes the rash is very mild, which makes it difficult for patients or their families or doctors to recall its existence. Arthritis is the most common manifestation and usually appears before the age of 10 years [5]. The arthritis is symmetrically, and the commonly involved joints are the wrists, metacarpal-phalangeal joints (MCP), proximal interphalangeal joints (PIP) of the hands and feet, and ankle joints. Meanwhile, the arthritis is frequently accompanied by synovial cysts and tenosynovitis. Long-term arthritis without effective treatment may even evolve into joint deformities. The uveitis in Blau syndrome is characterized by chronic bilateral panuveitis with multifocal chorioretinal lesions [31]. According to the literature [31], more than 50% of the patients have anterior and intermediate or posterior uveitis, while only approximately 30% of them have anterior uveitis only. Consistent with these findings, of the patients with ocular involvement in our cohort (6 patients), 33.3% (2 patients) presented with anterior uveitis, while 66.6% (4 patients) had intermediate and posterior segment uveitis. Thus, when patients have only arthritis and/or uveitis but no skin involvement or if the rash is ignored, these patients are easily misdiagnosed with JIA. However, there is a distinct difference in that only approximately 10–20% of JIA patients are associated with uveitis, and almost all of the uveitis cases in these patients are anterior uveitis [32, 33, 34]. In the present study, we describe a 20-year-old Chinese patient with Blau syndrome. She presented at 10 months of age with a skin rash, which was soon cured and never recurred. Therefore, when she was mistaken for having JIA at two years old, the history of rash was neglected by her parents and doctors. Her subsequent ocular involvement was also considered to be related to JIA. Regrettably, it was not until she was found to have hypertension when she was 17 years old that she was finally diagnosed with Blau syndrome. Hence, a complete past medical history review and continuous thinking about the correctness of diagnosis is essential when the previous diagnosis cannot explain all the manifestations and when the therapeutic effect is not ideal. In summary, for refractory JIA, it is necessary to inquire about any past history of rash and the patient’s family history to perform an ophthalmic examination to check whether there is evidence of uveitis. Genetic testing should be conducted if necessary.

In addition to the typical triad, the clinical phenotypes of Blau syndrome are complicated and can frequently present as different degrees of fever, liver and spleen lymph node enlargement, cardiac involvement, central nervous system involvement, and renal involvement. Blau syndrome complicated with vascular involvement is a rare manifestation, and the main manifestation is medium-and large-vessel arteritis. To our knowledge, only 18 patients have been reported in the English literature during the past 40 years. We describe a young Chinese girl who demonstrated the typical triad of Blau syndrome, consisting of polyarticular arthritis, uveitis, and rash. At the same time, there was evidence that she had right renal arteritis. According to previous studies [8, 9, 10, 11, 12, 13, 14, 15, 16, 17], all large, medium, and small blood vessels can be involved in Blau syndrome patients. It is noteworthy that the vast majority of the patients mentioned above had medium and larger vessel arteritis, which usually appears silently. Clinicians should be concerned about this potentially rare but serious manifestation in Blau syndrome patients. Therefore, careful examination of the patient’s peripheral pulse and measurement of the patient’s blood pressure at every regular visits are recommended, and if abnormalities are found, further evaluation for vascular involvement is advisable.

At present, various reports about different treatments for Blau syndrome exist [35]. However, there is no specific cure for this disease. Tumor necrosis factor-α (TNF-α) antagonists are effective against both Blau syndrome and Takayasu arteritis [36] and TNF-α is also a significant inflammatory factor in the NF-κB signaling pathway. Infliximab and adalimumab are used in the treatment of joint symptoms, and are also effective for improving eye symptoms [37, 38]. Analysis of the clinical data of Blau syndrome patients in Japan [3] showed that of the 26 patients treated with anti-TNF agents, only one was blind, and the blindness happened before the administration of biologics. In contrast, of the 14 patients who had not received biological treatment, 5 were blind. Similarly, the ocular symptoms of 6 patients treated with anti-TNF agents in our center improved to varying degrees and were maintained without deterioration. For patients with Blau syndrome, delayed or inappropriate treatment may lead to serious consequences, including joint contracture, blindness, and even fatal injury, which significantly affect the quality of life of the patient. Hence, early diagnosis and appropriate treatment may prevent or delay the occurrence of severe symptoms in patients to improve the patient’s quality of life.

## Conclusion

In conclusion, the typical triad of Blau syndrome usually appears discontinuously, and even in some patients, it may not all present because the patients are easily mistaken as having JIA. Therefore, when JIA cannot explain all the manifestations and the treatment effect is not ideal, we should rethink whether the diagnosis is correct. A detailed medical history inquiry and a careful physical examination are crucial, especially when the patient has a history of rash. Further examinations, such as genetic testing, are also necessary especially for infant-onset refractory JIA. Medium- and large-vessel arteritis is a rare clinical manifestation in Blau syndrome patients. Careful examination of the patient’s peripheral pulse and measurement of the patient’s blood pressure at every regular visit may be helpful in the early identification of Blau syndrome-arteritis. Ultimately, early diagnosis and appropriate treatment may prevent or delay the occurrence of severe symptoms in patients to improve the patient’s quality of life.

## Supplementary Information

**Additional file 1:** **Table S**. Clinical data of 8 children with blau syndrome in our center.

**Additional file 2:** **Fig. S1**. Current treatment of patients with Blau syndrome in our center. (A) Systemic therapy. We calculated all drugs of every patient in this graph. (B) Biologics. 6 cases were using anti-tumor necrosis factor (TNF) agents, including adalimumab in 4 cases and infliximab in 2cases. (C) Combination therapy of other therapies and biological agents. NSAIDs, non-steroidal anti-inflammatory drugs; MTX, methotrexate; PSL, prednisolone; Bio, biologics.

## Data Availability

Not applicable.

## References

[1] Miceli-Richard C, Lesage S, Rybojad M, Prieur AM, Manouvrier-Hanu S, Häfner R (2001). CARD15 mutations in Blau syndrome. Nat Genet.

[2] Blau EB (1985). Familial granulomatous arthritis, iritis, and rash. J Pediatr.

[3] Matsuda T, Kambe N, Ueki Y, Kanazawa N, Izawa K, Honda Y (2020). Clinical characteristics and treatment of 50 cases of Blau syndrome in Japan confirmed by genetic analysis of the NOD2 mutation. Ann Rheum Dis.

[4] Sfriso P, Caso F, Tognon S, Galozzi P, Gava A, Punzi L (2012). Blau syndrome, clinical and genetic aspects. Autoimmun Rev.

[5] Gattorno M, Federici S, Pelagatti MA, Caorsi R, Brisca G, Malattia C (2008). Diagnosis and management of autoinflammatory diseases in childhood. J Clin Immunol.

[6] Schwartz GJ, Muñoz A, Schneider MF, Mak RH, Kaskel F, Warady BA, Furth SL (2009). New equations to estimate GFR in children with CKD. J Am Soc Nephrol.

[7] Obrycki Ł, Sarnecki J, Lichosik M, Sopińska M, Placzyńska M, Stańczyk M (2022). Kidney length normative values in children aged 0–19 years - a multicenter study. Pediatr Nephrol.

[8] Rotenstein D, Gibbas DL, Majmudar B, Chastain EA (1982). Familial granulomatous arteritis with polyarthritis of juvenile onset. N Engl J Med.

[9] Gross KR, Malleson PN, Culham G, Lirenman DS, McCormick AQ, Petty RE (1986). Vasculopathy with renal artery stenosis in a child with sarcoidosis. J Pediatr.

[10] Rose CD, Eichenfield AH, Goldsmith DP, Athreya BH (1990). Early onset sarcoidosis with aortitis–"juvenile systemic granulomatosis?". J Rheumatol.

[11] Gedalia A, Shetty AK, Ward K, Correa H, Venters CL, Loe WA (1996). Abdominal aortic aneurysm associated with childhood sarcoidosis. J Rheumatol.

[12] Wang X, Kuivaniemi H, Bonavita G, Mutkus L, Mau U, Blau E (2002). CARD15 mutations in familial granulomatosis syndromes: a study of the original Blau syndrome kindred and other families with large-vessel arteritis and cranial neuropathy. Arthritis Rheum.

[13] Mourad F, Tang A (2010). Sinus of valsalva aneurysm in Blau’s syndrome. J Cardiothorac Surg.

[14] Khubchandani RP, Hasija R, Touitou I, Khemani C, Wouters CH, Rose CD (2012). Blau arteritis resembling Takayasu disease with a novel NOD2 mutation. J Rheumatol.

[15] Inoue Y, Kawaguchi Y, Shimojo N, Yamaguchi K, Morita Y, Nakano T (2013). A case of infantile Takayasu arteritis with a p.D382E NOD2 mutation: an unusual phenotype of Blau syndrome/early-onset sarcoidosis?. Mod Rheumatol..

[16] Amin SR, Pulido JS (2013). Retinal vasculitis, aneurysms, and neovascularization in Blau syndrome. JAMA Ophthalmol.

[17] Li C, Zhang J, Li S, Han T, Kuang W, Zhou Y (2017). Gene mutations and clinical phenotypes in Chinese children with Blau syndrome. Sci China Life Sci.

[18] Wouters CH, Maes A, Foley KP, Bertin J, Rose CD (2014). Blau syndrome, the prototypic auto-inflammatory granulomatous disease. Pediatr Rheumatol Online J.

[19] Tromp G, Kuivaniemi H, Raphael S, Ala-Kokko L, Christiano A, Considine E (1996). Genetic linkage of familial granulomatous inflammatory arthritis, skin rash, and uveitis to chromosome 16. Am J Hum Genet.

[20] Girardin SE, Boneca IG, Viala J, Chamaillard M, Labigne A, Thomas G (2003). Nod2 is a general sensor of peptidoglycan through muramyl dipeptide (MDP) detection. J Biol Chem.

[21] Chen G, Shaw MH, Kim Y-G, Nuñez G (2009). NOD-like receptors: role in innate immunity and inflammatory disease. Annu Rev Pathol.

[22] Al Nabhani Z, Dietrich G, Hugot J-P, Barreau F (2017). Nod2: The intestinal gate keeper. PLoS Pathog.

[23] Warner N, Burberry A, Franchi L, Kim Y-G, McDonald C, Sartor MA (2013). A genome-wide siRNA screen reveals positive and negative regulators of the NOD2 and NF-κB signaling pathways. Sci Signal..

[24] Ogura Y, Inohara N, Benito A, Chen FF, Yamaoka S, Nunez G (2001). Nod2, a Nod1/Apaf-1 family member that is restricted to monocytes and activates NF-kappaB. J Biol Chem.

[25] Tada H, Aiba S, Shibata K-I, Ohteki T, Takada H (2005). Synergistic effect of Nod1 and Nod2 agonists with toll-like receptor agonists on human dendritic cells to generate interleukin-12 and T helper type 1 cells. Infect Immun.

[26] Ogura Y, Lala S, Xin W, Smith E, Dowds TA, Chen FF (2003). Expression of NOD2 in Paneth cells: a possible link to Crohn’s ileitis. Gut.

[27] Rosé CD, Pans S, Casteels I, Anton J, Bader-Meunier B, Brissaud P (2015). Blau syndrome: cross-sectional data from a multicentre study of clinical, radiological and functional outcomes. Rheumatology (Oxford).

[28] Rose CD, Neven B, Wouters C (2014). Granulomatous inflammation: The overlap of immune deficiency and inflammation. Best Pract Res Clin Rheumatol.

[29] Rose CD (2017). Blau Syndrome: A Systemic Granulomatous Disease of Cutaneous Onset and Phenotypic Complexity. Pediatr Dermatol.

[30] Poline J, Fogel O, Pajot C, Miceli-Richard C, Rybojad M, Galeotti C (2020). Early-onset granulomatous arthritis, uveitis and skin rash: characterization of skin involvement in Blau syndrome. J Eur Acad Dermatol Venereol.

[31] Sarens IL, Casteels I, Anton J, Bader-Meunier B, Brissaud P, Chédeville G (2018). Blau Syndrome-Associated Uveitis: Preliminary Results From an International Prospective Interventional Case Series. Am J Ophthalmol.

[32] Saurenmann RK, Levin AV, Feldman BM, Rose JB, Laxer RM, Schneider R (2007). Prevalence, risk factors, and outcome of uveitis in juvenile idiopathic arthritis: a long-term followup study. Arthritis Rheum.

[33] Grassi A, Corona F, Casellato A, Carnelli V, Bardare M (2007). Prevalence and outcome of juvenile idiopathic arthritis-associated uveitis and relation to articular disease. J Rheumatol.

[34] Heiligenhaus A, Minden K, Föll D, Pleyer U (2015). Uveitis in juvenile idiopathic arthritis. Dtsch Arztebl Int.

[35] Matsuda T, Kambe N, Takimoto-Ito R, Ueki Y, Nakamizo S, Saito MK (2022). Potential Benefits of TNF Targeting Therapy in Blau Syndrome, a NOD2-Associated Systemic Autoinflammatory Granulomatosis. Front Immunol.

[36] Mekinian A, Biard L, Dagna L, Novikov P, Salvarani C, Espitia O (2022). Efficacy and safety of TNF-α antagonists and tocilizumab in Takayasu arteritis: multicentre retrospective study of 209 patients. Rheumatology (Oxford).

[37] Chen J, Luo Y, Zhao M, Wu D, Yang Y, Zhang W (2019). Effective treatment of TNFα inhibitors in Chinese patients with Blau syndrome. Arthritis Res Ther.

[38] Jindal AK, Pilania RK, Suri D, Gupta A, Gattorno M, Ceccherini I (2021). A young female with early onset arthritis, uveitis, hepatic, and renal granulomas: a clinical tryst with Blau syndrome over 20 years and case-based review. Rheumatol Int.

