# Supplementary material for: A Chinese girl of Blau syndrome with renal arteritis and a literature review
Source: Pediatr Rheumatol Online J. 2023 Mar 13;21:23. doi: 10.1186/s12969-023-00804-z (PMC10010039; doi:10.1186/s12969-023-00804-z)
Supplement: Supplementary file 1 — Additional file 1: Table S. Clinical data of 8 children with blau syndrome in our center. [file 12969_2023_804_MOESM1_ESM.docx]

**Table S.** Clinical data of 8 children with blau syndrome in our center.

| No | Gender | Time(year) | Age(year) | | | Initial symptom | Clinical manifeestation | | | | | | | | | Mutation |
| --- | --- | --- | --- | --- | --- | --- | --- | --- | --- | --- | --- | --- | --- | --- | --- | --- |
|  |  | diagnosis | Onset | First visit | Diagnosis |  | R | A | U | F | V | HBP | RI | LI | CI |  |
| 1 | Male | 2012 | 3 | 7.42 | 8.58 | R | * | * | * |  |  |  |  |  |  | R334W |
| 2 | Female | 2015 | 1 | 6.33 | 6.33 | A | * | * | * | * |  |  |  |  |  | R334W |
| 3 | Male | 2015 | 0.33 | 4.58 | 4.58 | R | * | * | * | * |  | * | * | * | * | NA |
| 4 | Male | 2017 | 0.5 | 2.08 | 2.08 | R | * | * |  |  |  |  |  |  |  | R334W |
| 5 | Male | 2017 | 1 | 8.58 | 8.58 | A |  | * | * |  |  |  |  |  |  | R334Q |
| 6 | Male | 2017 | 0.58 | 4.16 | 4.16 | R&A | * | * | * | * |  |  |  |  |  | M513A |
| 7 | Female | 2019 | 0.83 | 7.67 | 17.83 | R | * | * | * | * | * | * | * |  |  | M513T |
| 8 | Female | 2021 | 0.33 | 2.25 | 2.25 | R | * | * |  |  |  |  |  |  |  | R334W |

R:rash; A:arthritis; U:uveitis; F:fever; V:vasculitis; HBP:high blood pressure; RI:renal involvement; LI:liver involvement; CI:cardiac involvement;

*means that the patient had this clinical manifestation.
