# Supplementary figures and images for: A Chinese girl of Blau syndrome with renal arteritis and a literature review
Source: Pediatr Rheumatol Online J. 2023 Mar 13;21:23. doi: 10.1186/s12969-023-00804-z (PMC10010039; doi:10.1186/s12969-023-00804-z)

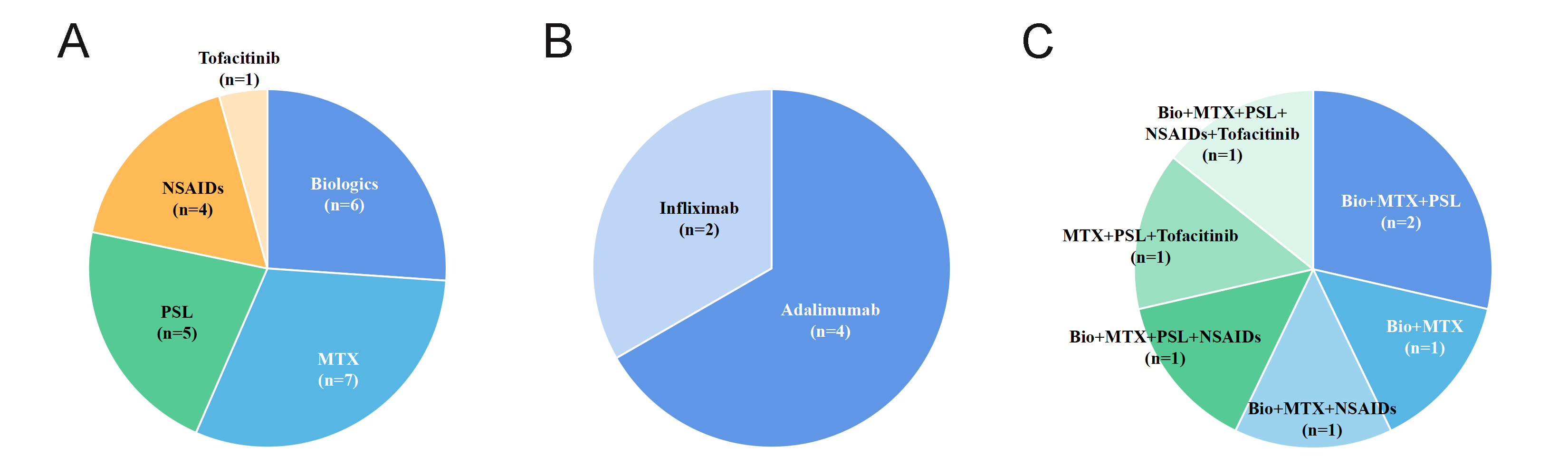

Supplement: Supplementary file 2 — Additional file 2: Fig. S1. Current treatment of patients with Blau syndrome in our center. (A) Systemic therapy. We calculated all drugs of every patient in this graph. (B) Biologics. 6 cases were using anti-tumor necrosis factor (TNF) agents, including adalimumab in 4 cases and infliximab in 2cases. (C) Combination therapy of other therapies and biological agents. NSAIDs, non-steroidal anti-inflammatory drugs; MTX, methotrexate; PSL, prednisolone; Bio, biologics. [file 12969_2023_804_MOESM2_ESM.tif]
